# Development of a Platform for Noncovalent Coupling of Full Antigens to Tobacco Etch Virus-Like Particles by Means of Coiled-Coil Oligomerization Motifs

**DOI:** 10.3390/molecules26154436

**Published:** 2021-07-23

**Authors:** Lorena Zapata-Cuellar, Jorge Gaona-Bernal, Carlos Alberto Manuel-Cabrera, Moisés Martínez-Velázquez, Carla Sánchez-Hernández, Darwin Elizondo-Quiroga, Tanya Amanda Camacho-Villegas, Abel Gutiérrez-Ortega

**Affiliations:** 1 Centro de Investigación y Asistencia en Tecnología y Diseño del Estado de Jalisco, Unidad de Biotecnología Médica y Farmacéutica, Normalistas 800, Colinas de la Normal, Guadalajara 44270, Mexico; zac.lorena@gmail.com (L.Z.-C.); caalmaca_07@hotmail.com (C.A.M.-C.); mmartinez@ciatej.mx (M.M.-V.); delizondo@ciatej.mx (D.E.-Q.); 2 Centro Universitario de Ciencias de la Salud, Departamento de Microbiología y Patología, Universidad de Guadalajara, Sierra Mojada 950, Independencia Oriente, Guadalajara 44340, Mexico; jorge.gaona@academicos.udg.mx; 3 Centro Universitario de Ciencias Biológicas y Agropecuarias, Departamento de Producción Agrícola, Universidad de Guadalajara, Carretera Guadalajara-Nogales km 15.5, Zapopan 45510, Mexico; carla.shernandez@academicos.udg.mx; 4 CONACYT-CIATEJ, Unidad de Biotecnología Médica y Farmacéutica, Normalistas 800, Colinas de la Normal, Guadalajara 44270, Mexico; tcamacho@ciatej.mx

**Keywords:** subunit vaccines, antigen carrier, virus-like particles, noncovalent coupling, heterodimerization motifs, antigen uptake

## Abstract

Virus-like particles are excellent inducers of the adaptive immune response of humans and are presently being used as scaffolds for the presentation of foreign peptides and antigens derived from infectious microorganisms for subunit vaccine development. The most common approaches for peptide and antigen presentation are translational fusions and chemical coupling, but some alternatives that seek to simplify the coupling process have been reported recently. In this work, an alternative platform for coupling full antigens to virus-like particles is presented. Heterodimerization motifs inserted in both Tobacco etch virus coat protein and green fluorescent protein directed the coupling process by simple mixing, and the obtained complexes were easily taken up by a macrophage cell line.

## 1. Introduction

The mammalian adaptive immune system has evolved to recognize viral structures in order to trigger a fast response when facing a viral attack. These viral structures have been named pathogen-associated molecular patterns, because of their highly ordered and repetitive nature, which allows for the efficient activation of B cells and also some innate immune responses [1]. Virus-like particles (VLPs) are particles devoid of genetic material that still conserve the symmetry of their infectious counterparts and, hence, their immunostimulatory properties. These VLPs are commonly formed by the spontaneous self-assembly of a coat or capsid protein (CP). Some remarkable examples of these VLPs, in terms of their importance to human health, are recombinant hepatitis B virus and papillomavirus vaccines, which contain hepatitis B surface antigen and papillomavirus major capsid protein L1, respectively, and have shown their protective efficacy against these viruses [2,3]. Given these special properties of VLPs, they have been extensively employed as scaffolds for enhancing the immunogenicity of foreign sequences derived from a wide variety of pathogens, serving as antigen or peptide carriers to the immune system for the development of subunit vaccines [4,5]. The original sources of these VLPs can be human [6,7,8,9,10,11], avian [12], bacterial [13] or plant viruses [14,15,16].

The most widely used approaches for fusing antigens or peptides to VLPs are translational fusions and chemical coupling. In the first case, the antigen/peptide coding sequence is introduced in-frame in any given site of the virus coat/capsid protein, generating chimeric VLPs that bear the foreign antigen/peptide. The main drawback of this approach is that the foreign amino acid sequence to be incorporated cannot be too long, generally no more than 20 amino residues, because the insertion of longer sequences may affect capsid protein structure to an extent that it compromises particle assembly [4]. In the case of chemical coupling, this approach basically consists of binding the foreign sequence to VLPs in a covalent fashion, taking advantage of lateral chains of certain amino acids that are surface exposed, such as lysine, cysteine, tyrosine, glutamic acid and aspartic acid, employing homo-/heterobifunctional crosslinkers for this purpose. Yet this approach allows the coupling of large sequences, even complete antigens, and it has some disadvantages, such as increasing the steps of the downstream process and, most importantly, the lack of efficacy and reproducibility of the coupling process, resulting in the formation of VLPs with a different degree of foreign antigen load, hardly reaching a 100% load [5].

Recently, some sophisticated methods for covalent coupling of foreign sequences to VLPs without the use of chemical crosslinkers have been developed. One method is based on the SpyCatcher-SpyTag technology, where two specific peptide sequences, one named SpyCatcher and the other named SpyTag, can form a spontaneous isopeptide bond between each other. SpyCatcher sequence was genetically fused to the amino terminus of the coat protein of phage AP205 and chimeric SpyCatcher-AP205 VLPs were formed. In the same way, the SpyTag sequence was genetically fused to the amino terminus of malaria antigens. SpyCatcher-AP205 VLPs and antigens were mixed together, and the spontaneous bond took place, leading to the acquisition of AP205 VLPs/malaria antigen complexes that induced specific antibody production in immunized mice [17]. Another method relies on bacterial sortase A (srtA), which is a transpeptidase able to bind a protein that bears a recognition motif to a polyG motif present in another protein. Sortase A recognition motif was added to the amino terminus of Papaya mosaic virus coat protein (PapMV CP), and resulting VLPs were linked to two different polyG peptides through recombinant sortase A. Both VLP/peptide complexes elicited an antibody response after their administration to mice [18].

Coiled-coil oligomerization motifs are widely distributed in nature and consist of a seven amino acid repeat in which two hydrophobic amino acids align at one side after the heptad folds into a helical conformation, and these hydrophobic amino acids are actually responsible for the oligomerization process [19]. Coiled-coil heterodimerization motifs have been previously employed for encapsulating green fluorescent protein (GFP) inside Cowpea chlorotic mottle virus (CCMV) VLPs in a two-step fashion, first binding CCMV CP monomers to GFP, both modified to have the complementary motifs, followed by VLP assembly by lowering the pH [20]. In this work, an alternative method for the noncovalent coupling of full antigens to VLPs based on coiled-coil heterodimerization motifs is reported. To achieve this, the complementary heterodimerization motifs were added to the open reading frames of Tobacco etch virus (TEV) CP and GFP, the proteins were expressed and purified separately and, finally, TEV VLPs were coupled to GFP by mixing, simplifying the process dramatically. It was demonstrated that the VLPs contributed to the uptake of the antigen by a murine macrophage cell line.

## 2. Results

### 2.1. Arrangement of Elements of Recombinant Proteins According to Structural Prediction

**Figure 1:**
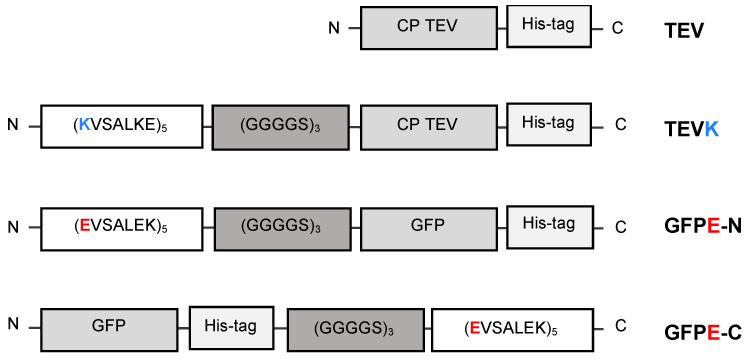
Graphical representation of viral coat protein (CP) and antigens. TEV: native Tobacco etch virus CP; TEVK: Tobacco etch virus CP having a K-coil motif; GFPE-N: green fluorescent protein (GFP) having an E-coil motif at N-terminus; GFPE-C: GFP having an E-coil motif at C-terminus. His-tag: six histidine tag; (GGGGS)_3_: flexible linker.

**Figure 2:**
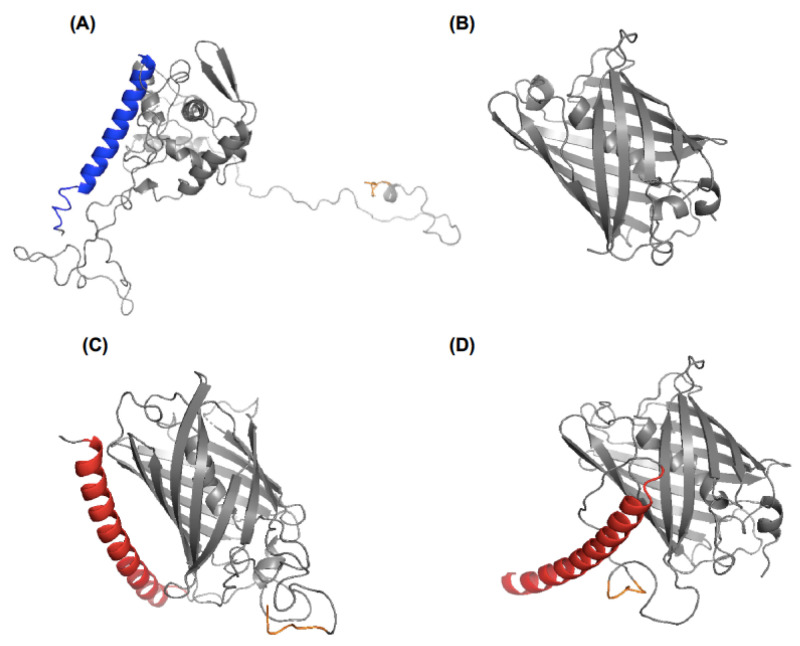
Protein structure prediction of viral coat protein and antigens. (**A**) TEVK protein with K-coil highlighted in blue. (**B**) Native GFP protein. (**C**) GFPE-N and (**D**) GFPE-C proteins with E-coil highlighted in red. His-tag is highlighted in orange.

The coiled-coil oligomerization motif sequences employed in this work for coupling VLPs to antigen were (KVSALKE)_5_ and (EVSALEK)_5_, designated as K- and E-coils, respectively [21]. The organization of coiled-coil motifs, flexible spacer, protein and His-tag in the amino acid sequences TEVK, GFPE-N and GFPE-C are shown in Figure 1. Previous works suggest that the N-terminus of TEV CP is displayed on the surface of both viral particles and VLPs [22,23]. Additionally, a His-tag in the C-terminus of *E. coli*-expressed TEV CP enhances VLP recovery [24]. The disposition of K-coil, E-coil and His-tag in the tertiary structure was observed in the protein models obtained by multitemplate modeling and ab initio with Phyre2 (Figure 2). For TEVK, 75% of the residues were modeled with >90% confidence, the CP sequence shared 65% identity with c6t34I (Chain I, Turnip mosaic virus coat protein structure) [25] and the same percentage with c5odvB (Chain B, Watermelon mosaic virus coat protein structure) [26], both belonging to potyviruses. K-coil was modeled with 83% of identity with the template c3tq2A (Chain A, KE1, merohedral twinning in protein crystals revealed a new synthetic three-helix bundle motif) [27]. For GFPE-N and GFPE-C, 91% of the residues were modeled with >90% confidence finding 98% of identity between GFP sequence and the template c5fguA (Chain A, structure of sda1 nuclease apoprotein as an egfp fixed-arm fusion) [28]. E-coil had a 71% identity with the template c3w93C (Chain C, crystal structure analysis of the synthetic gcn4 ester coiled-coil peptide) [29]. According to the models, coiled-coil motifs and His-tags were exposed in the protein surface except for His-tag of GFPE-C, which was hidden between E-coil and GFP.

### 2.2. Expression of Recombinant TEVK, GFPE-N and GFPE-C Proteins in E. coli

**Figure 3:**
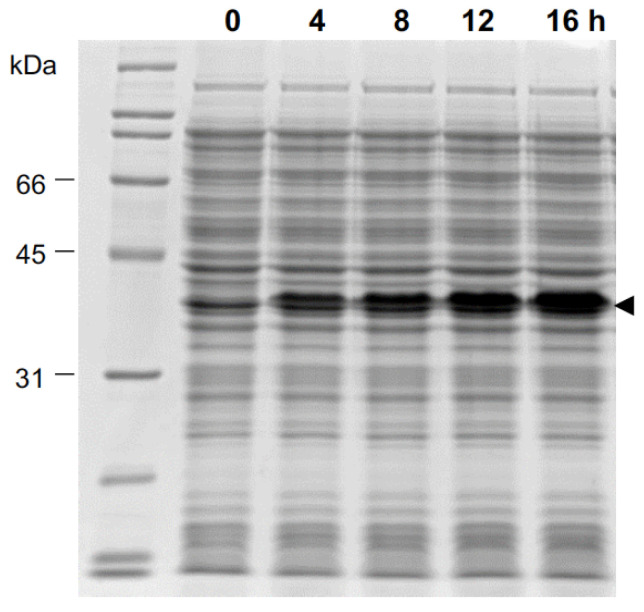
TEVK protein expression time course in *E. coli*. Cells were grown in Terrific broth (modified) and protein expression was induced with 1.0 mM IPTG at 20 °C and analyzed by SDS-PAGE at 0, 4, 8, 12 and 16 h.

Successful protein expression was evidenced by SDS-PAGE by the presence of intense bands that correspond to the theoretical weight of 35.8 kDa for TEVK and 32.7 kDa for GFPE-N and GFPE-C. The best parameters for TEVK protein expression were 20 °C, 1.0 mM IPTG and 16 h in Terrific broth (modified) (Figure 3). The expression time course with 0.5 mM and 2.0 mM IPTG is shown in Figure S1 of Supplementary Material. Expression of GFPE-N and GFPE-C proteins was achieved at 30 °C, 5 h after the induction with 0.5 mM IPTG.

**Figure 4:**
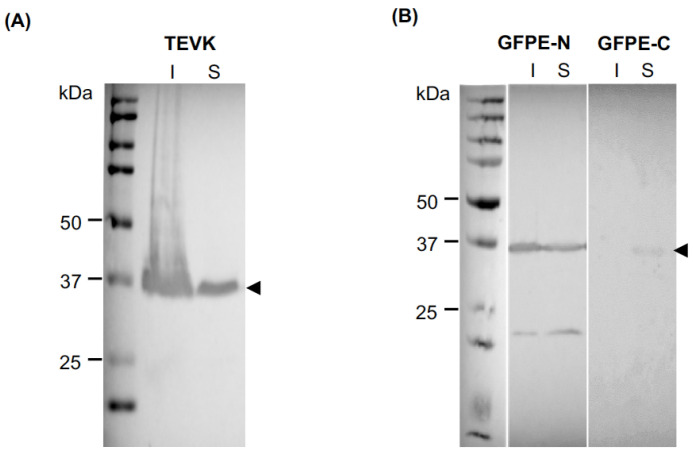
Western blot of (**A**) TEVK and (**B**) GFPE-N and GFPE-C proteins in bacterial lysates. I: insoluble fraction. S: soluble fraction.

To assess protein accumulation in the soluble and insoluble fraction of *E. coli* lysates, both fractions were analyzed by Western blot (Figure 4). It was found that approximately 50% of TEVK and GFPE-N was present in the soluble fraction, which was chosen for protein purification, thus avoiding the use of chaotropic or denaturing agents. On the other hand, a lower expression of GFPE-C was observed in the soluble fraction, but these levels were enough for the purification process.

### 2.3. Purification of Recombinant Proteins

**Figure 5:**
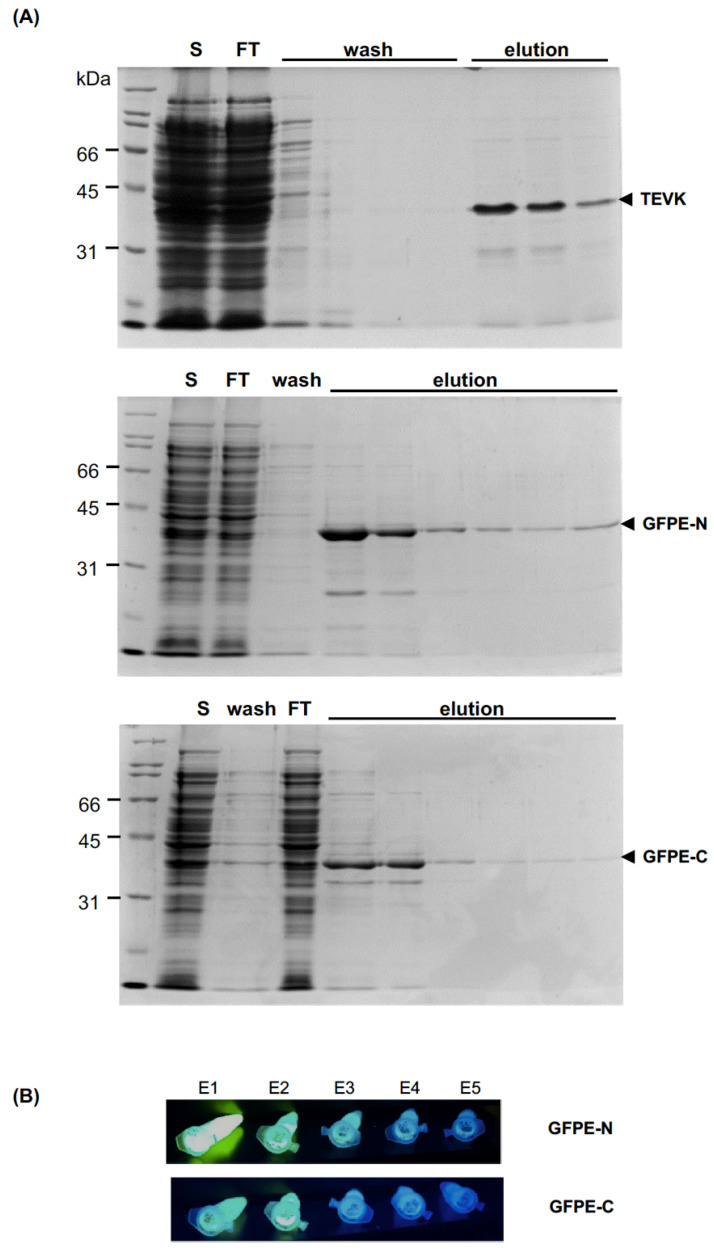
Purification of TEVK, GFPE-N and GFPE-C proteins by IMAC. (**A**) Analysis of the purification process by SDS-PAGE. S: Soluble fraction of bacterial lysate; FT: soluble fraction flowthrough; wash: wash solution flowthrough; elution: elution fractions. (**B**) Fluorescence of GFPE-N and GFPE-C versions in the elution fractions.

The recombinant proteins were purified by immobilized metal affinity chromatography (IMAC). TEVK was bound to the column; however, increased protein loss was observed using concentrations greater than 5mM imidazole in the binding process. Washing with buffers containing increasing concentrations of NaCl provided the appropriate ionic environment to keep TEVK and eliminate *E. coli* proteins from the column. Finally, elution with 500 mM imidazole yielded protein with a high level of purity in the first three elution fractions (Figure 5A). GFPE-N and GFPE-C proteins were purified using a shorter process. GFPE-N was bound to the column, adjusting soluble fraction to 10 mM imidazole and GFPE-C required a concentration of up to 5 mM or less. A greater amount of GFPE-C was lost in comparison to GFPE-N during the washing process. The protein model shows that the His-tag is partially hidden in the tertiary structure of GFPE-C, and this could reduce the affinity of the protein to nickel ions immobilized in the purification column, causing the protein to flow through the column during the binding and washing steps of the process. Both proteins were mostly recovered in the first two elution fractions (Figure 5A). The elution fractions of both versions of GFP evidenced the conservation of the fluorescence (Figure 5B).

### 2.4. Observation of TEVK VLPs in Presence of GFP Proteins with Complementary Coiled-Coil Motifs

**Figure 6:**
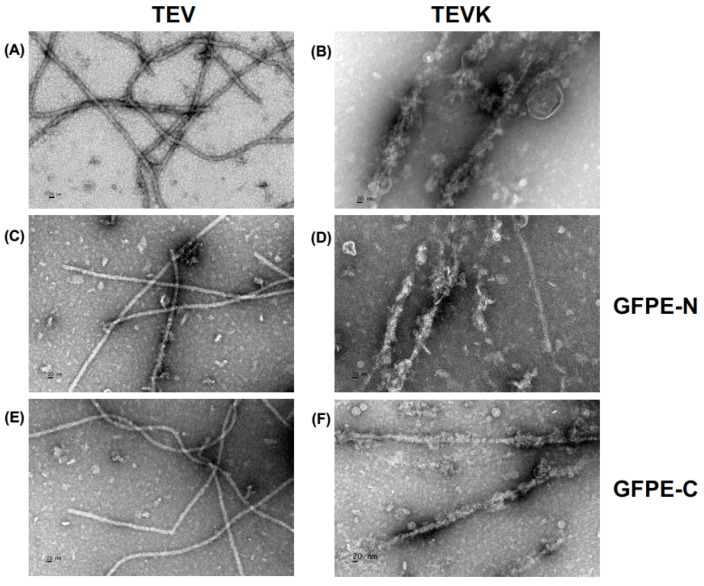
VLP assembly confirmation by transmission electron microscopy. (**A**) Native TEV CP. (**B**) TEVK. (**C**) Native TEV mixed with GFPE-N protein. (**D**) TEVK mixed with GFPE-N protein. (**E**) Native TEV mixed with GFPE-C protein. (**F**) TEVK mixed with GFPE-C protein. Scale bar: 20 nm.

The ability of TEVK to form VLPs was analyzed by transmission electron microscopy (TEM), comparing it with native TEV VLPs. In both cases, filamentous particles similar to potyviral particles are observed. The observation of particles of up to 1 um demonstrated the utility of the method to obtain VLPs. Previous reports demonstrated that the addition of foreign sequences in the amino terminus of potyviral CPs did not hamper VLP formation [30,31,32]. It was confirmed that the TEVK protein retains its ability to self-assemble into VLPs in the presence of GFPE-N or GFPE-C. Characteristically, TEVK VLPs showed less surface uniformity, suggesting protuberances due to the possible interaction between CP monomers and the K-coil motifs or, when it corresponds, the heterogeneous binding of GFPE in the surface of VLP (Figure 6). The surface of the VLPs resembles supramolecular structures formed by cholera toxin B-subunit proteins having coiled-coil motifs [33].

### 2.5. Interaction of Chimeric VLPs and Complete Antigens Containing K-Coil and E-Coil Interaction Motifs

**Figure 7:**
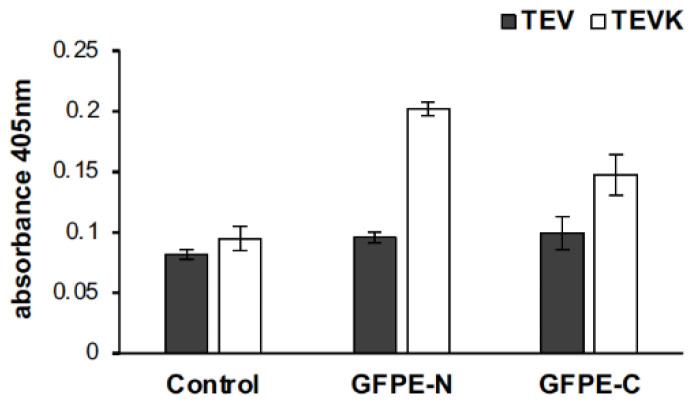
Interaction analysis of TEVK VLPs with GFPE-N and GFPE-C proteins by ELISA. GFPE-N or GFPE-C versions were used as capture antigens. Anti-TEV alkaline phosphatase-labeled antibody was used to measure the interacting VLPs.

The interaction of the GFPE-N or GFPE-C proteins with TEVK VLPs was evidenced by incubating these proteins with TEVK VLPs coupled to an anti-TEV antibody in an ELISA assay. The absorbance showed that the interaction of GFPE-N or GFPE-C proteins incubated with native TEV VLPs that do not possess the interaction motif is negligible and comparable with the negative control in which the VLP/anti-TEV complex was placed in the absence of antigen. Conversely, the binding of the antigens was notably higher when treated with TEVK VLPs, which bear the interaction motif (Figure 7). This suggests the interaction of GFPE-N and GFPE-C proteins to VLPs driven by complementary E- and K-coils. Furthermore, the absorbance was higher when GFPE-N was used, because this combination offers a low steric hindrance for the parallel binding of the complementary interaction motifs present in the VLPs and GFP. Parallel binding of the complementary motifs has been confirmed by Minten and collaborators [34].

### 2.6. Chimeric VLPs with K-Coil Stimulate Internalization of Antigens in Murine Macrophages

A fundamental activity in vaccine models is the phagocytosis of antigens by cells of the innate immune system. The repetitive presentation of antigens, as in the case of VLPs, stimulates their recognition by cells of the immune system, including antigen-presenting cells (APCs), such as macrophages or dendritic cells.

**Figure 8:**
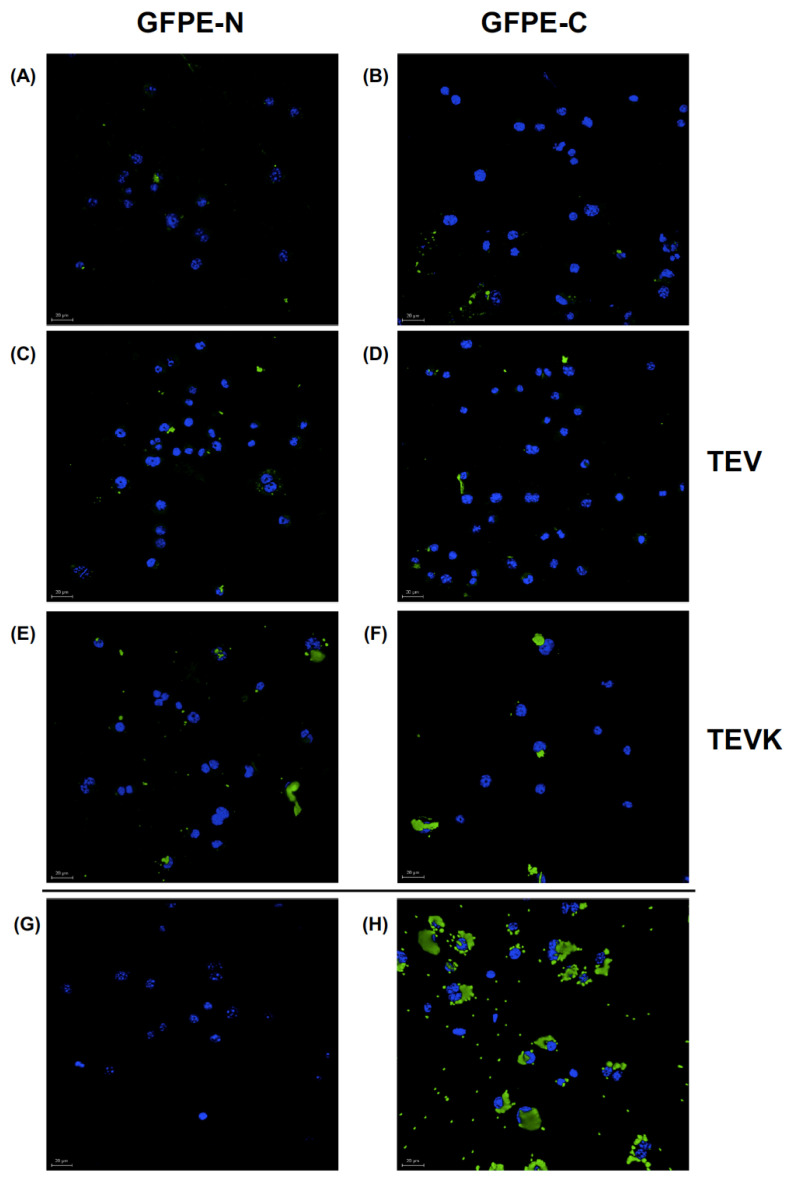
Phagocytosis of antigens coupled to VLPs by murine macrophages. Confocal microscopy of cells incubated with (**A**) GFPE-N or (**B**) GFPE-C alone, (**C**) GFPE-N or (**D**) GFPE-C mixed with TEV VLPs, (**E**) GFPE-N or (**F**) GFPE-C mixed with TEVK VLPs. (**G**) Untreated cells and (**H**) Cells incubated with latex beads coupled to FITC as positive control. Nuclei stained with DAPI. Scale bar: 20 μm.

The internalization of soluble antigens alone and in the presence of VLPs with the K-coil motif in murine macrophages was compared. Phagocytosis of the GFPE-N or GFPE-C proteins as reporter antigens was analyzed by confocal microscopy. The DAPI-stained cell nuclei and the intracellular location of FITC-labeled latex beads were seen as indicative of the phagocytic ability of the cells used in the assay. Phagocytosis of both versions of GFP was found. When comparing the internalization patterns of the GFPE-N protein with the TEV VLP/GFPE-N mixture and the TEVK VLP/GFPE-N mixture, we found that the TEVK VLP/GFPE-N mixture stimulates localized phagocytosis of GFPE-N protein compared to the TEV VLPs without the interaction motif. Similarly, a comparison of the phagocytosis patterns of the GFPE-C protein indicates that the TEVK VLP/GFPE-C combination stimulates phagocytosis. In both cases, the dispersion of small intracellular complexes of GFPE-N or GFPE-C showed that intracellular localization of the antigen is favored in the presence of VLP compared to soluble protein alone, but when administered with TEVK VLPs, larger fluorescent complexes are observed, indicating an improvement in the uptake of antigens by murine macrophages driven by the carrying effect of TEVK VLPs (Figure 8).

## 3. Discussion

In this work, an alternative approach to couple full-length antigens to VLPs based on coiled-coil protein interaction motifs is reported. Besides eliminating the need for chemical crosslinkers, it allows the binding of the antigens to VLPs in the presence of a widely used buffer that is highly compatible with living organisms. This way, the VLP/antigen complexes can be administered for immunization without any further process right after they have been generated. Furthermore, the strength and stability of the complexes formed by this approach need to be determined, as the immunostimulatory properties of the VLPs rely on these two parameters for vaccination purposes. This could represent a drawback in comparison to the SpyCatcher-SpyTag and sortase A approaches [17,18], where stable covalent bonds are formed between VLPs and antigen. Nonetheless, to overcome this disadvantage, cysteines could be added to the complementary coiled-coil sequences so that covalent disulfide bonds are formed under mildly oxidizing conditions by close contact between these residues after motif interaction [35]. It is suggested that the addition of coiled-coil protein interaction motifs to both the TEV CP and GFP versions did not severely affect their folding, as evidenced by TEM, where TEV CP chimeric VLPs were observed and fluorescence emission of GFP versions under UV light. However, for a deeper characterization of the VLPs, cryoelectron microscopy must be carried out. It can be noticed that TEVK VLP filaments alone are less uniform than TEVK VLPs mixed with GFPE-N/C proteins. Apparently, GFP binding stabilizes the VLP structure, but there is no explanation for this. Previous works demonstrate that potyviral CPs can accommodate large foreign sequences in amino terminus and yet assemble into VLPs. For example, VLPs were observed by TEM after fusing rubredoxin protein (71 amino acids long) to the amino terminus of Potato virus Y CP [31]. Finally, the TEV VLPs enhanced the uptake of bound proteins by murine macrophages, as evidenced by confocal microscopy. Antigen uptake and APC stimulation are key steps for the induction of a strong adaptive immune response, which is the foundation of vaccination. Recognition of viral particles in the surface of APCs induces lipid raft aggregates, triggering APC stimulation. It has been previously demonstrated that PapMV interacts with the cell surface of mouse macrophages and dendritic cells, inducing lipid raft aggregation and eliciting a strong immune response [36]. The antigen uptake experiment suggests that the VLP/antigen complex will elicit a strong immune response in a live animal model, so an animal immunization protocol with this complex definitively should be carried out to assess the potential of this technology.

## 4. Materials and Methods

### 4.1. Sequence Design and Structural Prediction

The TEV CP (GenBank: JX512813.1) was modified, including a seven-residue repeat (KVSALKE)_5_ in the N-terminus. This repeat forms a coiled-coil named K [21]. Between the TEV CP and the K-coil, we included a flexible spacer sequence that consists of a five-residue repeat (GGGGS)_3_. The resultant protein was named TEVK. In parallel, two GFP proteins that possess an E-coil, which consists of the sequence (EVSALEK)_5_ that forms a coiled-coil complementary to the K motif [21], were designed. The sequence (EVSALEK)_5_ was included in the N-terminus of GFPE-N protein, and a flexible spacer sequence (GGGGS)_3_ was included between the protein and the E-coil. Conversely, the GFPE-C protein contains the sequence (EVSALEK)_5_ in the C-terminus, and a flexible spacer sequence (GGGGS)_3_ was included between the protein and the E-coil. With the amino acid sequence of each protein design, a prediction of the 3D structures was made by multi-template modeling and a simplified ab initio folding using the intensive mode of Phyre V2.0 online server (http://www.sbg.bio.ic.ac.uk/phyre2/html/page.cgi?id=index, accessed on 8 April 2020) [37]. 

The theoretical physicochemical characteristics of the proteins were obtained with the tool “Protparam” from the EXPASY portal (http://web.expasy.org/protparam/, accessed on 8 April 2020).

### 4.2. Construction of Expression Plasmids and Cell Transformation

Based on the amino acid sequences of the modeled proteins, three genes optimized for expression in *Escherichia coli* (*E. coli*) were synthesized (GenScript, Piscataway, NJ, USA), adding NcoI and XhoI cleavage sites at the 5’and 3’ ends, respectively. For the construction of expression plasmids, the synthetic sequences were digested with the NcoI and XhoI restriction enzymes (New England Biolabs, Ipswich, MA, USA), purified with the MinElute Gel Extraction Kit (Qiagen, Germantown, MD, USA) and ligated to the bacterial expression plasmid pET28a+ (Merck-Millipore, Burlington, MA, USA) with T4 DNA ligase (Promega, Madison, WI, USA). The ligation products were used to transform electrocompetent *E. coli* One Shot Top10 cells (Thermo Fisher Scientific, Waltham, MA, USA), and cells were spread over Petri dishes containing semisolid LB agar medium (Sigma-Aldrich, Saint Louis, MO, USA) supplemented with 50 μg/mL kanamycin sulfate (Thermo Fisher Scientific, Waltham, MA, USA). Plasmid DNA was isolated from some transformant colonies with the GeneJet Plasmid Miniprep kit (Thermo Fisher Scientific, Waltham, MA, USA) and analyzed by restriction pattern with XbaI and XhoI enzymes (Promega, Madison, WI, USA) to confirm the new plasmid constructs pET28-TEVK, pET28-GFPE-N and pET28-GFPE-C. The confirmed constructs were used to transform chemically competent *E. coli* One Shot BL21Star (DE3) cells (Thermo Fisher Scientific, Waltham, MA, USA). The strain expressing native TEV CP (without K-coil) was previously generated [24].

### 4.3. Protein Expression in E. coli and Verification by Western Blot

Four-milliliter cultures from fresh colonies containing the plasmids pET28-TEVK, pET28-GFPE-N and pET28-GFPE-C were prepared overnight, and 2 mL from TEVK culture was placed in a 1000 mL glass Erlenmeyer flask with 200 mL of Terrific broth (modified) medium (Sigma-Aldrich, Saint Louis, MO, USA) for the expression of TEVK, while Lennox LB medium (Sigma-Aldrich, Saint Louis, MO, USA) was used for the expression of GFPE-N and GFPE-C. All cultures were incubated at 37 °C and 250 rpm until an OD_600_ of 1.0 was reached. Expression of TEVK protein was induced with 0.5 mM, 1.0 mM and 2.0 mM of isopropyl-β-D-1-thiogalactopyranoside (IPTG) (Promega, Madison, WI, USA) for 16 h at 20 °C. Expression of GFPE-N and GFPE-C proteins was induced with 0.5 mM of the same inducer for 10 h at 30 °C. All cells were collected by centrifugation (4 °C and 5000× *g* for 25 min) and stored at −20 °C until processing. 

The cells were resuspended in 1/10 of the culture’s volume using lysis buffer (20 mM Tris-HCl pH 8.0, 500 mM NaCl) and lysed with a Microson XL-2000 sonicator (Farmingdale, NY, USA) by 20 rounds of 30 s. The soluble fraction was separated from the cell debris by centrifugation of total protein lysate (4 °C and 5000× *g* for 25 min) and 20 μg of total protein, soluble and insoluble fraction (as determined with Bradford reagent) (Sigma-Aldrich, Saint Louis, MO, USA) were analyzed by SDS-PAGE and stained with Coomassie Brilliant Blue (Biorad, Hercules, CA, USA), using the molecular weight marker Unstained SDS-PAGE Standards, Broad Range (Biorad, Hercules, CA, USA). For immunodetection of the proteins, Western blot was performed using anti-6xHis Tag monoclonal antibody (Roche, Basel, Switzerland) at a 1:500 dilution in 5% skim milk and goat anti-mouse secondary antibody coupled to peroxidase (R&D systems, Minneapolis, MN, USA) at a 1:1000 dilution, using 4-chloro-1-naphthol substrate (Biorad, Hercules, CA, USA) for color development.

### 4.4. Purification of VLPs and Soluble Proteins by IMAC

The soluble fraction of TEVK was adjusted to 5 mM imidazole and loaded into a 1 mL His Trap HP IMAC column (GE-Healthcare, Chicago, IL, USA). Twenty column volumes (CV) were injected into the column, followed by a wash with 30 CV with Wash Solution 1 (20 mM Tris pH 8.0, 200 mM NaCl, 40 mM imidazole), 20 CV of Wash Solution 2 (20 mM Tris pH 8.0, 400 mM NaCl, 40 mM imidazole), 20 CV of Wash Solution 3 (20 mM Tris pH 8.0, 600 mM NaCl, 40 mM imidazole) and 20 CV of Wash Solution 4 (20 mM Tris pH 8.0, 800 mM NaCl, 40 mM imidazole). Finally, the protein was eluted with 10 CV of elution buffer (20 mM Tris pH 8.0, 500 mM NaCl, 500 mM imidazole) and collected in 1 mL fractions.

The GFPE-N and GFPE-C proteins were purified injecting 10 CV of the soluble fraction with imidazole (10 mM for GFPE-N and 5 mM for GFPE-C) into the purification column, followed by 20 CV of wash solution (20 mM Tris pH 8.0, 1 M NaCl, 40 mM imidazole) for GFPE-N and 10 CV for GFPE-C. Finally, the proteins were eluted with 10 CV of elution buffer (20 mM Tris pH 8.0, 500 mM NaCl, 500 mM imidazole) and collected in 1 mL fractions.

In all cases, the elution fractions with the highest protein concentration were pooled and buffer exchange was carried out with 20 mM Tris pH 8.0, 500 mM NaCl or PBS pH 7.4, 500 mM sodium chloride using a 10 kDa MWCO Amicon Ultra-15 filtration centrifugal unit (Merck-Millipore, Burlington, MA, USA).

### 4.5. Electron Microscopy

TEVK protein and TEV CP samples were prepared at concentrations of 200 μg/mL. Additionally, mixtures of these proteins with GFPE-N and GFPE-C in a ~1:1 molar ratio with a final concentration of 200 μg/mL of each protein were generated. All samples were prepared in buffer solution (20 mM Tris pH 8.0, 500 mM NaCl, 20 mM EDTA), placed on formvar-coated copper grids and stained with 2% uranyl acetate solution. The grids were analyzed by TEM using a JEM-100C (JEOL, Tokyo, Japan) equipment.

### 4.6. VLP/Antigen Interaction Analysis

The assay was performed on a 96-well plate coated with 1 µg of GFPE-N or GFPE-C antigen per well (20 ng/μL) and 100 mM carbonate buffer pH 9.6 as background control. Immobilization was carried out at 4 °C overnight. Subsequently, each well was blocked with 250 µL of 3% BSA (Sigma-Aldrich, Saint Louis, MO, USA) in PBS for 1 h at 37 °C. Thereafter, three washes with 0.05% Tween-20 in PBS (wash buffer) were performed. TEV or TEVK VLPs were mixed with anti-TEV antibody (Agdia, Elkhart, IN, USA) in 1:100 dilution using PBS/1% BSA as a diluent, and each mixture was incubated at 37 °C/200 rpm for 2.5 h. The antibody/VLP mixture was added to the wells in triplicate, calculating 1 µg of each VLP per well. As control, anti-TEV antibody alone was added in 1:100 dilution to antigen-coated wells (data not shown). To allow interaction, the plate was incubated at 37 °C for 1 h. Subsequently, the plate was washed three times with wash buffer and once with PBS. Finally, 50 µL of Alkaline Phosphatase Yellow (pNPP) Liquid Substrate System for ELISA (Sigma-Aldrich, Saint Louis, MO, USA) was added to each well, and absorbance was read at 405 nm.

### 4.7. Internalization Analysis of VLP/Antigen Complexes vs. Soluble Antigens in Murine Macrophages

A phagocytosis assay was performed using the murine macrophage cell line RAW 264.7. The cell line was maintained at 37 °C with 5% CO_2_ in MEM culture medium (Hyclone, Logan, UT, USA) supplemented with 10% FBS (Thermo Fisher Scientific, Waltham, MA, USA). The assay was performed in 12-well culture plates with a coverslip placed at the bottom of each well, and 3 × 10^5^ cells were seeded in 800 μL of culture medium per well. The following was added to the cells: 200 μL of PBS as a negative control, 1 × 10^6^ one µm diameter latex beads coupled to FITC (Molecular Probes, Eugene, OR, USA) as a positive control, 10 μg of TEV VLPs, 10 μg of the TEVK VLPs, 10 μg of GFPE-C antigen, 10 μg of GFPE-N antigen, TEV VLPs/GFPE-C (10 μg each), TEV VLPs/GFPE-N (10 μg each), TEVK VLPs/GFPE-C (10 μg each) and TEVK VLPs/GFPE-N (10 μg each). All protein formulations were prepared in PBS, 500 mM NaCl, pH 7.4 and added to each well. The cells were incubated at 37 °C, 5% CO_2_ for periods of 45 min, then the samples were washed twice with PBS, fixed with methanol previously cooled to −20 °C and washed twice with PBS. The coverslips were mounted with Vectashield mounting medium with DAPI (Vector Laboratories, Burlingame, CA, USA), and the samples were visualized on a Leica DM5500 Q confocal microscope (Leica, Wetzlar, Germany).

## 5. Conclusions

Here, an alternative approach for coupling VLPs to full-length antigens by simple mixing through the use of protein interaction motifs is reported. It was predicted that the interaction motifs added to both the VLP-forming CP and model antigen did not affect their correct folding. The VLPs stimulated antigen uptake by murine macrophages in vitro. Taken together, these findings suggest that this VLP/antigen coupling approach could be useful for the development of subunit vaccines.

## Data Availability

Data is contained within the article.

## Supplementary Materials

The following are available online, Figure S1: TEVK protein expression at two different concentrations of IPTG. (**A**) 0.5 mM IPTG (**B**) 2.0 mM IPTG in Terrific Broth (modified). Protein expression was analyzed by SDS-PAGE at 0, 4, 8, 12, and 16 h.

## References

[1] Bachmann M.F., Jennings G.T. (2010). Vaccine delivery: A matter of size, geometry, kinetics and molecular patterns. Nat. Rev. Immunol..

[2] Zanetti A.R., van Damme P., Shouval D. (2008). The global impact of vaccination against hepatitis B: A historical overview. Vaccine.

[3] Dadar M., Chakraborty S., Dhama K., Prasad M., Khandia R., Hassan S., Munjal A., Tiwari R., Karthik K., Kumar D. (2018). Advances in designing and developing vaccines, drugs and therapeutic approaches to counter human papilloma virus. Front. Immunol..

[4] Plummer E.M., Manchester M. (2011). Viral nanoparticles and virus-like particles: Platforms for contemporary vaccine design. Wiley Interdiscip. Rev. Nanomed. Nanobiotechnol..

[5] Bárcena J., Blanco E., Mateu M.G. (2013). Design of novel vaccines based on virus-like particles or chimeric virions. Structure and Physics of Viruses.

[6] Berkower I., Raymond M., Muller J., Spadaccini A., Aberdeen A. (2004). Assembly, structure, and antigenic properties of virus-like particles rich in HIV-1 envelope gp120. Virology.

[7] Ye L., Lin J., Sun Y., Bennouna S., Lo M., Wu Q., Bu Z., Pulendran B., Compans R.W., Yang C. (2006). Ebola virus-like particles produced in insect cells exhibit dendritic cell stimulating activity and induce neutralizing antibodies. Virology.

[8] Warfield K.L., Swenson D.L., Olinger G.G., Kalina W.V., Aman M.J., Bavari S. (2007). Ebola virus-like particle-based vaccine protects nonhuman primates against lethal Ebola virus challenge. J. Infect. Dis..

[9] Perrone L.A., Ahmad A., Veguilla V., Lu X., Smith G., Katz J.M., Pushko P., Tumpey T.M. (2009). Intranasal vaccination with 1918 influenza virus-like particles protects mice and ferrets from lethal 1918 and H5N1 influenza virus challenge. J. Virol..

[10] Huber B., Schellenbacher C., Jindra C., Fink D., Shafti-Keramat S., Kirnbauer R. (2015). A chimeric 18L1-45RG1 virus-like particle vaccine cross-protects against oncogenic alpha-7 human papillomavirus types. PLoS ONE.

[11] Chen S., Zheng D., Li C., Zhang W., Xu W., Liu X., Fang F., Chen Z. (2015). Protection against multiple subtypes of influenza viruses by virus-like particle vaccines based on a hemagglutinin conserved epitope. Biomed. Res. Int..

[12] Liu Y.V., Massare M.J., Pearce M.B., Sun X., Belser J.A., Maines T.R., Creager H.M., Glenn G.M., Pushko P., Smith G.E. (2015). Recombinant virus-like particles elicit protective immunity against avian influenza A(H7N9) virus infection in ferrets. Vaccine.

[13] Pastori C., Tudor D., Diomede L., Drillet A.S., Jegerlehner A., Röhn T.A., Bomsel M., Lopalco L. (2012). Virus like particle based strategy to elicit HIV-protective antibodies to the alpha-helic regions of gp41. Virology.

[14] Marusic C., Rizza P., Lattanzi L., Mancini C., Spada M., Belardelli F., Benvenuto E., Capone I. (2001). Chimeric plant virus particles as immunogens for inducing murine and human immune responses against human immunodeficiency virus type 1. J. Virol..

[15] Denis J., Acosta-Ramirez E., Zhao Y., Hamelin M.E., Koukavica I., Baz M., Abed Y., Savard C., Pare C., Macias C.L. (2008). Development of a universal influenza A vaccine based on the M2e peptide fused to the papaya mosaic virus (PapMV) vaccine platform. Vaccine.

[16] Lico C., Mancini C., Italiani P., Betti C., Boraschi D., Benvenuto E., Baschieri S. (2009). Plant-produced potato virus X chimeric particles displaying an influenza virus-derived peptide activate specific CD8+ T cells in mice. Vaccine.

[17] Brune K.D., Leneghan D.B., Brian I.J., Ishizuka A.S., Bachmann M.F., Draper S.J., Biswas S., Howarth M. (2016). Plug-and-Display: Decoration of virus-like particles via isopeptide bonds for modular immunization. Sci. Rep..

[18] Thérien A., Bédard M., Carignan D., Rioux G., Gauthier-Landry L., Laliberté-Gagné M.È., Bolduc M., Savard P., Leclerc D. (2017). A versatile papaya mosaic virus (PapMV) vaccine platform based on sortase-mediated antigen coupling. J. Nanobiotechnol..

[19] Dong H., Hartgerink J.D. (2006). Short homodimeric and heterodimeric coiled coils. Biomacromolecules.

[20] Minten I.J., Hendriks L.J.A., Nolte R.J.M., Cornelissen J.J.L.M. (2009). Controlled encapsulation of multiple proteins in virus capsids. J. Am. Chem. Soc..

[21] Litowski J.R., Hodges R.S. (2002). Designing heterodimeric two-stranded α-helical coiled-coils. Effects of hydrophobicity and α-helical propensity on protein folding, stability, and specificity. J. Biol. Chem..

[22] Shukla D.D., Strike P.M., Tracy S.L., Gough K.H., Ward C.W. (1988). The N and C termini of the coat proteins of potyviruses are surface-located and the N terminus contains the major virus-specific epitopes. J. Gen. Virol..

[23] Manuel-Cabrera C.A., Márquez-Aguirre A., Rodolfo H.G., Ortiz-Lazareno P.C., Chavez-Calvillo G., Carrillo-Tripp M., Silva-Rosales L., Gutiérrez-Ortega A. (2012). Immune response to a potyvirus with exposed amino groups available for chemical conjugation. Virol. J..

[24] Manuel-Cabrera C.A., Vallejo-Cardona A.A., Padilla-Camberos E., Hernández-Gutiérrez R., Herrera-Rodríguez S.E., Gutiérrez-Ortega A. (2016). Self-assembly of hexahistidine-tagged tobacco etch virus capsid protein into microfilaments that induce IgG2-specific response against a soluble porcine reproductive and respiratory syndrome virus chimeric protein. Virol. J..

[25] Cuesta R., Yuste-Calvo C., Gil-Cartón D., Sánchez F., Ponz F., Valle M. (2019). Structure of Turnip mosaic virus and its viral-like particles. Sci. Rep..

[26] Zamora M., Méndez-López E., Agirrezabala X., Cuesta R., Lavín J.L., Sánchez-Pina M.A., Aranda M.A., Valle M. (2017). Potyvirus virion structure shows conserved protein fold and RNA binding site in ssRNA viruses. Sci. Adv..

[27] Geremia S., De March M. (2012). RCSB PDB—3TQ2: Merohedral Twinning in Protein Crystals Revealed a New Synthetic Three Helix Bundle Motif. Ph.D. Thesis.

[28] Moon A.F., Krahn J.M., Lu X., Cuneo M.J., Pedersen L.C. (2016). Structural characterization of the virulence factor Sda1 nuclease from Streptococcus pyogenes. Nucleic Acids Res..

[29] Dadon Z., Samiappan M., Shahar A., Zarivach R., Ashkenasy G. (2013). A high-resolution structure that provides insight into coiled-coil thiodepsipeptide dynamic chemistry. Angew. Chem. Int. Ed..

[30] Fernández-Fernández M.R., Martínez-Torrecuadrada J.L., Casal J.I., García J.A. (1998). Development of an antigen presentation sytem based on plum pox potyvirus. FEBS Lett..

[31] Kalnciema I., Skrastina D., Ose V., Pumpens P., Zeltins A. (2012). Potato virus Y-like particles as a new carrier for the presentation of foreign protein stretches. Mol. Biotechnol..

[32] Aguilera B.E., Chávez-Calvillo G., Elizondo-Quiroga D., Jimenez-García M.N., Carrillo-Tripp M., Silva-Rosales L., Hernández-Gutiérrez R., Gutiérrez-Ortega A. (2017). Porcine circovirus type 2 protective epitope densely carried by chimeric papaya ringspot virus–like particles expressed in Escherichia coli as a cost-effective vaccine manufacture alternative. Biotechnol. Appl. Biochem..

[33] Ross J.F., Wildsmith G.C., Johnson M., Hurdiss D.L., Hollingsworth K., Thompson R.F., Mosayebi M., Trinh C.H., Paci E., Pearson A.R. (2019). Directed assembly of homopentameric cholera toxin B-subunit proteins into higher-order structures using coiled-coil appendages. J. Am. Chem. Soc..

[34] IMinten J., Nolte R.J.M., Cornelissen J.J.L.M. (2010). Complex assembly behavior during the encapsulation of green fluorescent protein analogs in virus derived protein capsules. Macromol. Biosci..

[35] Wuo M.G., Mahon A.B., Arora P.S. (2015). An Effective Strategy for Stabilizing Minimal Coiled Coil Mimetics. J. Am. Chem. Soc..

[36] Acosta-Ramírez E., Pérez-Flores R., Majeau N., Pastelin-Palacios R., Gil-Cruz C., Ramírez-Saldaña M., Manjarrez-Orduño N., Cervantes-Barragán L., Santos-Argumedo L., Flores-Romo L. (2008). Translating innate response into long-lasting antibody response by the intrinsic antigen-adjuvant properties of Papaya mosaic virus. Immunology.

[37] Kelley L.A., Mezulis S., Yates C.M., Wass M.N., Sternberg M.J.E. (2015). The Phyre2 web portal for protein modeling, prediction and análisis. Nat. Protoc..

