# Supplementary material for: Development of a Platform for Noncovalent Coupling of Full Antigens to Tobacco Etch Virus-Like Particles by Means of Coiled-Coil Oligomerization Motifs
Source: Molecules. 2021 Jul 23;26(15):4436. doi: 10.3390/molecules26154436 (PMC8348948; doi:10.3390/molecules26154436)
Supplement: Supplementary file 1 [file molecules-26-04436-s001.zip › molecules-1294205-supplementary.pdf]

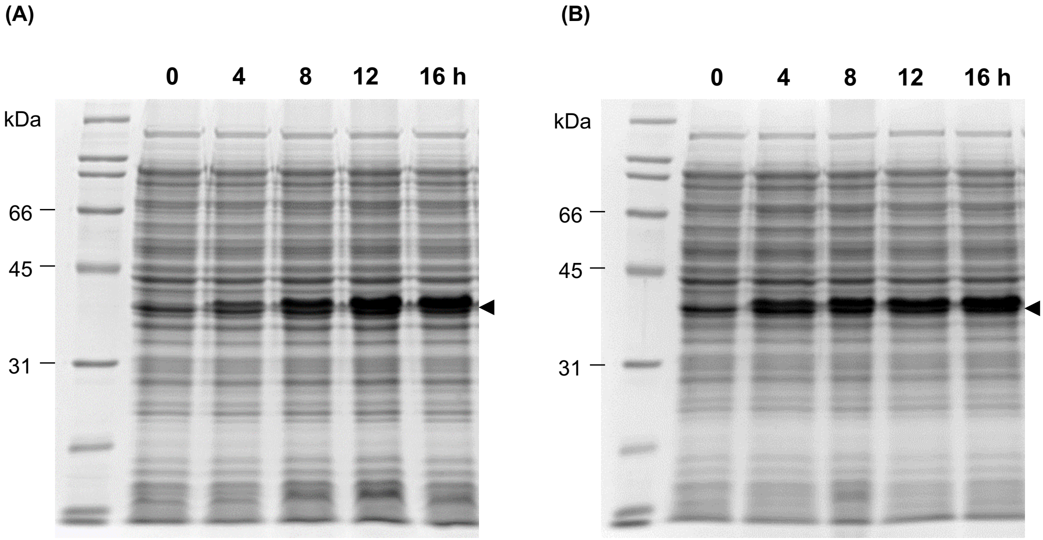

Figure S1: TEVK protein expression at two different concentrations of IPTG. (**A**) 0.5 mM IPTG (**B**) 2.0 mM IPTG in Terrific Broth (modified). Protein expression was analyzed by SDS-PAGE at 0, 4, 8, 12, and 16 h.
